# The Composite Task Reveals Stronger Holistic Processing in Children than Adults for Child Faces

**DOI:** 10.1371/journal.pone.0006460

**Published:** 2009-07-29

**Authors:** Tirta Susilo, Kate Crookes, Elinor McKone, Hannah Turner

**Affiliations:** Department of Psychology, Australian National University, Canberra, Australian Capital Territory, Australia; National Institute of Mental Health, United States of America

## Abstract

**Background:**

While own-age faces have been reported to be better recognized than other-age faces, the underlying cause of this phenomenon remains unclear. One potential cause is holistic face processing, a special kind of perceptual and cognitive processing reserved for perceiving upright faces. Previous studies have indeed found that adults show stronger holistic processing when looking at adult faces compared to child faces, but whether a similar own-age bias exists in children remains to be shown.

**Methodology/Principal Findings:**

Here we used the composite face task – a standard test of holistic face processing – to investigate if, for child faces, holistic processing is stronger for children than adults. Results showed child participants (8–13 years) had a larger composite effect than adult participants (22–65 years).

**Conclusions/Significance:**

Our finding suggests that differences in strength of holistic processing may underlie the own-age bias on recognition memory. We discuss the origin of own-age biases in terms of relative experience, face-space tuning, and social categorization.

## Introduction

Several studies have suggested that own-age faces are better recognised than other-age faces, a phenomenon usually termed the *other-age effect* or *own-age bias* [1]–[3]. As with the more established *other-race effect* – better recognition memory for own-race relative to other-race faces (for review, see [4]) – the own-age effect suggests that the sensitivity of the human visual system in recognising individual faces is related in some way to the frequency with which that type of face is encountered in the everyday environment.

Exactly what lies behind these recognition memory biases, however, is less understood. One plausible candidate is *holistic/configural processing*, a special mechanism reserved for perceiving upright faces that integrates information (including spacing between features) from across the entire face at a perceptual level [5]–[8]. In the other-race effect literature, it has been demonstrated that holistic processing is indeed stronger for own-race than other-race faces, at least for Caucasian participants [9], [10].

Two recent studies have found an own-age bias on holistic processing in adult participants: specifically, for adults with no special recent experience with children, holistic processing was stronger for adult faces than child faces [11], [12]. In children, however, previous studies have failed to find an own-age bias on holistic processing [13], [14], despite other demonstrations of an own-age bias on recognition memory [1], [15].

It is notable that behind the apparently conflicting results are different experimental paradigms. The studies which found the own-age bias [11], [12] used Young et al's composite face task [8], whereas the studies which found no own-age bias [13], [14] used Tanaka and Farah's part-whole task [7] and Tanaka and Sengco's part-in-spacing-changed-whole task [16]. Here we aimed to contribute to the question of whether an own-age bias can be found in children by using the composite face task, and comparing the size of the composite effect in children and adults for child face stimuli. It is well established that children show a composite effect with adult faces [17]–[19], and also with familiar child faces [17], but to our knowledge there have been no previous tests of the composite effect for children with unfamiliar child faces, and no comparisons of the size of the composite effect for child faces (familiar or unfamiliar) between children and adults.

**Figure 1:**
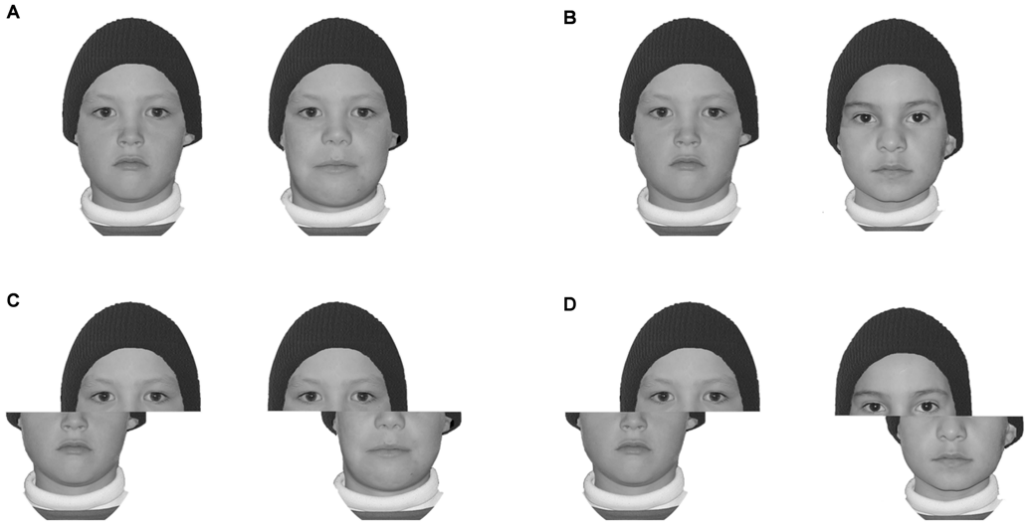
Examples pairs of our composite face stimuli. (A) same-aligned (SA), (B) different-aligned (DA), (C) same-misaligned (SM), and (D) different-misaligned (DM). The composite effect can be seen by comparing (A) with (C): in both cases, the two top half faces are physically identical, but, while this is easy to see in the misaligned version, it is difficult to see in the aligned version because perceptual integration of the whole face makes the top half appear different depending on which bottom half it is combined with. To tap the strength of this illusion, the composite effect is measured as the reduction in accuracy for “same” decisions in (A) as compared to (C).

In the present study, if children show stronger holistic processing for own-age faces, then we predict a larger composite effect for children relative to adults. We measured the strength of the composite illusion using the standard same-different procedure (see Figure 1); this is the version of the task used in one of the studies that previously demonstrated an own-age bias on holistic processing in adults [11].

## Methods

### Participants

The 48 participants comprised 20 children (age range 8–13 years, *M* = 10 years, 9 female) and 28 adults (age range 22–65 years, *M* = 44 years, 26 female). Participants were twins attending the 2009 Australian Twins Plus Festival in Sydney. (We were not interested here in twins *per se*; the present data was a serendipitous finding from a larger ongoing twin project). All were volunteers (no payment), naïve to the purpose of the study, had normal or corrected-to-normal vision, and were Caucasian (the same race as the face stimuli). Adults were a random sample of professions (i.e., as a group, they were not selected to be school teachers [11], [12] or otherwise to have any specific expertise with children).

### Stimuli

The original faces (i.e., from which composites were constructed) were from a database of photographs taken in Australia [20]. They were front view greyscale photographs of 48 unfamiliar Caucasian male children, with neutral expression, mostly aged 6–7 years with a few 5 year-olds. Importantly, while the specific age of the face stimuli was not matched to the age of our child participants, (a) primary school in Australia covers the age range of 5 to 12 years and so most of our child participants would see multiple 6–7 year-olds every day at school; and (b) an own-age bias on *recognition memory* for these particular faces has been previously demonstrated for children, in which the own-age advantage was as strong in older children (10–11 year-olds) as in a closely age-matched group (5–6 year-olds) [15]. A black ski-cap and white turtleneck collar were pasted onto each face to remove hair and clothing identity cues.

Figure 1 shows composite face examples. Each original face was divided horizontally below the eyes. The composite faces were created by joining the top half of one individual with the bottom half of a different individual. The top halves were always kept physically identical to the original; the size of the bottom halves was adjusted where necessary (to fit the corresponding top half). Misaligned faces were created by offsetting the top and bottom halves by half a face width. Half of the misaligned faces were offset to the left, the other half to the right. Aligned faces subtended a viewing angle of 6.3° horizontal by 9.7° vertical, and misaligned faces 8.6° horizontal by 9.7° vertical. Faces were presented against a grey background. All manipulations were done using Adobe Photoshop 5.5.

The composite faces were paired either as “same” or “different”; “same” pairs always had identical top-halves, “different” pairs always had different top-halves. The bottom halves for all pairs were always different. The result was four kinds of composite pairs: same-aligned (SA), same-misaligned (SM), different-aligned (DA), and different-misaligned (DM).

There were 30 different bottom halves and 30 different top halves. In the SA condition each top half was used once and each bottom half was used twice (because two different bottom halves were required for each pair of same top halves). The exact same composite combinations were used in the SM condition. In the DA condition each top half was shown once, 14 of the bottom halves were shown twice and two were shown once. The same composite combinations were used in the DM condition.

There were 90 composite face pairs in total, comprising 30 SA, 30 SM, 15 DA, and 15 DM pairs. The greater number of “same” pairs were intended to increase the proportion of trials relevant to the final analysis (a procedure used previously, [9], [18]), because the composite score was defined in the standard way, namely as the accuracy difference between the same-aligned (SA) and same-misaligned (SM) trials [9], [18], [21]–[23]. Only “same” trials contribute to the measure of the composite effect because, while holistic processing makes a clear prediction that “same” responses should be more difficult for aligned than misaligned trials (Figure 1), it makes no prediction of the direction of the alignment effect for “different” trials (the direction will depend on the similarity of to-be-ignored bottom halves, (see [23]), with the result that analysis of “different” accuracy and d' are meaningless (for further discussion, see [24]).

### Procedure

Each participant was tested using a CRT-screen iMac computer in an open function room with several other activities occurring around. They were seated at a distance of approximately 40 cm from the computer screen without any chin rest.

Participants were instructed to focus on the two top-halves of the sequentially presented pairs of faces and respond as to whether they were the same or different via a keyboard. It was emphasized that they were to ignore the bottom half of the face.

The 90 trials (30 SA, 30 SM, 15 DA, 15 DM) were displayed in random order. Each trial started with the presentation of the first face for 500 ms, followed by a blank screen for 400 ms and the second face for 500 ms. Each face appeared randomly in one of four different positions on the screen (up left, up right, down left and down right at 5° of eccentricity from the center of the screen). Following a blank screen of 400 ms, the question “Were the two top-halves same or different?” appeared until response. The next trial followed after 400 ms. Five practice trials were given.

The task was designed to measure accuracy. There were no instructions to respond quickly (and indeed we observed that some participants took their time, meaning that no analysis of reaction times was possible). We did not aim to measure reaction times because (a) it is inappropriate to measure reaction times when accuracy is set to be well below ceiling, and (b) baseline reaction times will inevitably vary substantially across ages from 8 to 65 years, affecting validity of comparison of the size of the composite effect across age [15].

## Results

**Figure 2:**
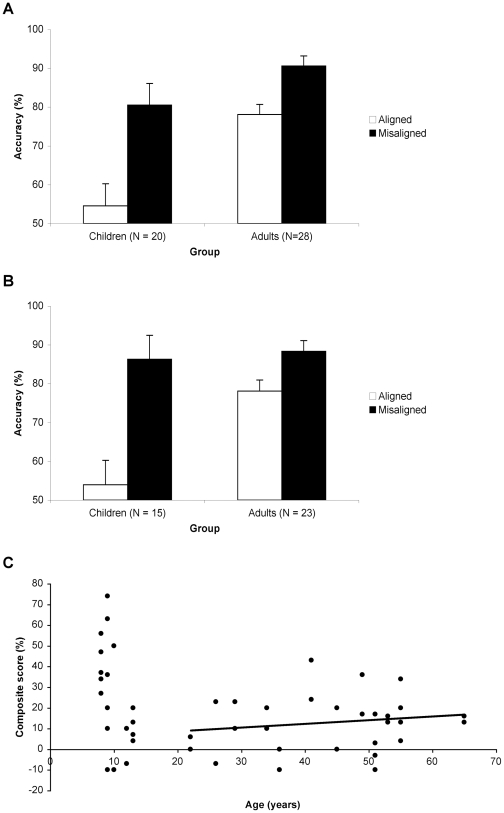
Results. (A) Accuracy (% correct matches) for same-aligned and same-misaligned trials in the full sample, showing a larger composite effect in children than adults. Error bars show ±1 SEM of the composite effect score, as appropriate for the within-subject comparison of aligned and misaligned. (B) The same result holds for a subset of participants for whom “baseline” performance in the control misaligned condition was matched across age groups. (C) Scatterplot of age versus composite score, with best linear fit for the adults, showing no age-related decline in holistic processing in older adults.

Results are shown in Figure 2 (also see Table 1). We analysed the 30 same-aligned and 30 same-misaligned trials only. The composite score was calculated as accuracy for misaligned trials *minus* accuracy for aligned trials.

**Table 1:** Mean accuracies for same and different trials.

Data Set	Group	N	Aligned Accuracy (%)	Misaligned Accuracy (%)	Composite Score (%) (Misaligned Accuracy - Aligned Accuracy)
Full	Children	20	54.6 (5.2)	80.5 (3)	25.9 (5.6)
	Adults	28	78.1 (2.6)	90.6 (1.5)	12.5 (2.6)
Baseline-matched	Children	15	54 (6.8)	86.3 (2)	32.3 (6.2)
	Adults	23	78.1 (2.9)	88.3 (1.5)	10.5 (2.8)

(A) Mean accuracies (% correct matches) for aligned and misaligned conditions in the full and baseline-matched datasets of the same trials. (B) Mean accuracies for aligned and misaligned conditions of the different trials. SEM in brackets.

Considering results for the full sample (Figure 2A), statistical analysis showed greater variability in composite scores for children than adults (Levene's test for equality of variances, *F* = 10.32, *p* = .002). Thus, in comparing the mean composite effect across groups, degrees of freedom were adjusted appropriately (using Welch-Satterthwaite equation via the “equal variances not assumed” output in SPSS). The composite effect was significantly larger in children (25.9%) than in adults (12.5%), *t*(27.21) = 2.22, *p*<.05, indicating stronger holistic processing for children than adults when looking at child faces.

We then conducted several analyses to confirm that this result could not be attributed to spurious confounds with other variables. First, we noted that the accuracy in the “baseline” misaligned condition was higher for adults than children, *t*(46) = 3.32, *p*<.01. Although there is no indication in Figure 2A that aligned-misaligned differences were affected by proximity to ceiling (or floor), we have argued elsewhere that much caution needs to be used when effects are compared across age groups in the presence of baseline differences [15]. Thus, we also analysed results from a *baseline-matched subset* (Figure 2B), created by removing the data of the 5 children with the lowest and 5 adults with the highest *misaligned* scores. Misaligned scores for the two groups were successfully matched (86.3% vs. 88.3%), *t*<1, but children's mean composite score (32.3%) was still larger than adults' (10.5%), *t*(19.77) = 3.31, *p*<.01 (Levene's test for equality of variances, *F* = 6.84, *p* = .013). This analysis demonstrates that our finding of stronger holistic processing for own-age faces in children is not due to mismatched baseline performance of the two groups.

Second, it is possible that the age-group difference could be attributed to the fact that our adult sample included a very wide range of ages. If there were a reduction in holistic processing with aging (e.g., after, say, 50 years of age), or if holistic processing for child faces continued to reduce in strength the longer the time since the participant had been a child, the comparison of the composite effect in children with that in the adult group could be affected. However, Figure 2C provides a scatterplot of exact age against the composite effect score (for the full sample), and shows that there was no decline across the adult age range. Statistical analysis confirmed that, within adults, there was no correlation between age and composite score, *r*(28) = .17, *p* = .398.

Third, because our participants were twins, their performance might not have been totally independent from one another (as we have assumed above in conducting independent-samples t-tests). We therefore conducted a 2×2 ANOVA with twin pairs as a repeated measure factor and age group as a between-subject factor. The main effect of age group was again found to be significant, *F*(1,22) = 37.82, *p*<.01, confirming a larger composite effect in children than in adults.

Finally, before turning to theoretical interpretation, it is necessary to dispose of one last potential limitation in our study. This is the unequal distribution of gender across age. In the child group, 45% of participants (9 out of 20) were female, whereas in the adult group, 93% (26 out of 28) were female. This raises the possibility that the weaker holistic processing observed in adults may have something to do with being female. However, the literature suggests that it is females who have better recognition memory with faces in general [25]. More relevant to our study, females' superior recognition ability extends to child faces [26], and this sex difference is also present in children [27]. Therefore, if anything, the prediction of our study would have been stronger holistic processing for adult participants, where there was a higher proportion of females. Yet our findings were the opposite, in that it was the child participants who showed stronger holistic processing.

## Discussion

Our results are novel in several ways. First, they provide the first demonstration that children show a composite effect for unfamiliar child faces. Second, they provide the first comparison of the size of the composite effect for child faces across child and adult participants, and thus provide the first evidence that the composite effect is larger in the former case. Finally, they provide the first comparison of the composite effect across participant age, for *any* age of face, that avoids problems associated with restriction of range due to ceiling effects in adults (see next section for details).

### An own-age bias or a larger composite effect in children for faces of all ages?

We have shown that children have a larger composite effect than adults for child faces. Our preferred interpretation is that this arises from an own-age bias on holistic processing in child participants, and thus complements earlier demonstrations of own-age biases on holistic processing in adult participants [11], [12].

However, given that we did not test an adult face set, there is an alternative possible interpretation, namely that children might show a larger composite effect that adults for *all* face ages. Previous data [18], [19] do seem to show, at first glance, that children have a stronger composite effect than adults even when tested with adult faces: the size of the composite effect in de Heering et al [18] was 19% for children (aged 4–6 years) and 7% for adults; and in Macchi Cassia et al [19], with a slightly different way of creating the composites, it was 11% for children (aged 3–5 years) and 5% for adults. In both studies, however, there was a methodological issue that prevents valid comparison of the size of the composite effect across age groups. Specifically, there was a substantial difference in overall performance between age groups such that adult participants performed close to ceiling (the average of same-aligned and same-misaligned was 92% [18] and 93% [19]) while children's performance was placed nicely in the middle of the 2AFC 50–100 range (82% [18] and 77% [19]). This means that, while both studies [18], [19] provide compelling and theoretically important evidence that young children show strong composite effects, the claim of a stronger composite effect in children than adults could be due simply to a restriction-of-range problem in adults. This interpretation is directly supported by two studies with adult participants [28], [29], taken from the same laboratory as the de Heering et al [18] study. These studies used composite stimuli constructed in the same way as in de Heering et al [18] (i.e., with a small vertical gap between the top and bottom halves) but set task difficulty so as to avoid ceiling effects in adults (2AFC task with average of aligned and misaligned performance 86% [28] and 78% [29]). Under these circumstances, the size of the composite effect for adults was 15% [28] and 22% [29]; this is very comparable in size to that found for children in de Heering et al (19%) [18].

In addition to this evidence, there is a second reason to think that there should be no differences between the size of the composite effect between children and adults for adult faces. The composite effect is a measure of holistic processing. The disproportionate inversion effect (the amount by which the inversion-reduction in memory for faces exceeds the inversion-reduction in memory for objects) is another measure of holistic processing. For adult faces, Crookes and McKone [15] found that the disproportionate inversion effect was the same size in children and adults. Also, again using adult faces, both Crookes and McKone [15] and Carey [30] found the size of the inversion effect for faces itself was the same size in children and adults. Crucially, both studies matched baseline performance across age groups. These inversion results therefore make a strong case that holistic processing is *not* larger in children than adults for adult faces.

Taking all findings together, we believe the most probable interpretation of the present result is that it represents an own-age bias in children for children's faces. We acknowledge, however, that to date there have been no studies that allow direct valid comparison of the size of the composite effect across children and adults for adult faces, and thus it remains possible (although we believe unlikely) that future studies could demonstrate that children show larger composite effects for all face types.

### Comparison with part-whole studies in children

Our composite effect results are in conflict with the two previous part-whole studies [13], [14], both of which tested child faces and did not find that holistic processing was stronger in children than adults. What is the origin of this conflict? We see two possibilities.

First, it may be (again) due to the presence of baseline differences between age groups in the earlier studies, which placed scores sufficiently close to ceiling (in adults) or floor (in children) so that range to show the holistic processing effect tested might have been restricted in one or other age group. In Pellicano and Rhodes [13], the average of the two conditions compared to calculate holistic processing (part and whole) was nicely in the middle of the 2AFC accuracy scale for adults (80%), but was low enough to perhaps produce a restriction-of-range problem in children (63%). Correspondingly, children showed a nonsignificant trend towards *less* holistic processing than adults (i.e., the reverse direction to the present study). In Pellicano et al [14], there was the opposite problem of potential restriction-of-range in adults (average across whole and part-in-spacing-changed-whole conditions = 90%), but not children (average = 71%); and, correspondingly, children showed a nonsignificant trend towards *more* holistic processing than adults (i.e., the same direction as the present study). Thus, in failing to equate baselines, the methodology of [13] and [14] may have masked any own-age bias.

The second possibility is that task itself matters (part-whole [13] and part-in-spacing-changed-whole [14]) versus the composite effect (present study). That is, while the part-whole and composite effects are both widely accepted as good measures of holistic processing, there may be some poorly understood difference between them that could produce genuine differences in results for child faces between the two tasks. In the absence of part-whole studies that have equated baseline performance across age groups, however, it would premature to draw any such conclusion at this stage.

### Origins of an own-age bias on holistic processing

Overall, we suggest that our results in children complement those of previous papers in adults to make a strong case that holistic processing can be influenced by own-age effects, just as it is influenced by own-race effects. This implies that differences in holistic processing for different face types may be an important variable driving corresponding differences in recognition memory for own-age as well as own-race faces.

We next consider the possible cause of an own-age bias on holistic processing. Presumably, this relates in some way to the amount of (recent) visual experience participants have with different face types: two recent studies have found that preschool teachers showed stronger holistic processing for child faces than did ordinary (“child-face-novice”) adults [11], [12]. (Another intriguing aspect of both studies is that while preschool teachers showed stronger holistic processing for child faces, they also showed weaker holistic processing for adult faces than the novice group. On a speculative note, this seems to indicate some kind of trade-off between the use of holistic processing for own-age and other-age faces. Perhaps holistic face processing capacity is limited such that it is automatically deployed more for the most commonly encountered or socially important face type. Our present data are silent with respect to this issue, since we did not test our child participants with adult faces. This speculation predicts that, in future studies, children with more visual experience of, or social interest toward, adult faces would show stronger holistic processing with adult faces than child faces.) Similarly, our own child participants (most of whom saw 6–7 year old faces at school every day) would have had greater recent experience with children's faces than did our adult participants (who were unselected for profession).

It remains an open question, however, as to whether the relationship of holistic processing to experience is direct or indirect. There may be a direct effect on the tuning of perceptual processing mechanisms. By analogy, dimensions of face-space are commonly argued to be tuned by recent exposure to match the “face diet” to which one has been exposed (e.g., when explaining adaptation aftereffects for faces; [31]).

Alternatively, it may be that there is no direct causal effect of experience on holistic processing, but that the relationship may arise indirectly via the correlation between experience and social categorisation, social interest, and/or attention given to difference face types. Face memory has been shown to be reduced by social outgroup categorisation [32] and, in the race field, it has been shown that strength of holistic processing can be altered merely by changing the perceived race group of an ambiguous-race face stimulus (an Asian-Caucasian morph; [33]). It may be that similar social effects contribute to other-age effects. In explaining previous findings in adults, it may be that people who choose to become preschool teachers are likely to be socially interested in children (and to not spontaneously categorise them as social outgroup members). Similarly, in our own study, the children may well have treated child faces as ingroup members more so than did the adults. Indeed, if the 8–13 year old children differed *amongst themselves* in how strongly they categorised 5–7 year old face stimuli as ingroup members, this could explain why our child group showed not only a greater mean composite score but also higher *variance* in composite scores than our adult group.

Of course, these two proposals (direct and indirect influences) are not necessarily mutually exclusive. It could be that the own-age bias on holistic processing is caused by some interaction between the amount, quality, and recency of visual experience with a face type, tuning of perceptual mechanisms, and social categorization.

### Development of the “special” aspect of face recognition

Finally, our results have theoretical implications for a topic quite different than own-age bias, namely the development of the “special” aspect of face recognition across childhood. It is now widely agreed that holistic processing is qualitatively present in very young children (i.e., all the standard phenomena have been demonstrated at 4–5 years, including composite, part-whole, inversion, sensitivity to spacing between facial features [13], [15], [18], [34]). There have been different recent views, however, about whether holistic processing remains immature until late in childhood in the sense that it is *quantitatively* weaker in children than in adults [15], [35]: results of many studies do suggest this on a *prima facie* basis [35] but we have argued elsewhere [15] that the findings of increases in holistic processing effects with age are unreliable due to substantial baseline changes across age groups. Our present study joins an emerging literature arguing that holistic processing is in fact quantitatively mature earlier rather than later (for review see [15]). In fact, our findings show that it is possible for children's holistic processing to be *stronger* than adults'.
